# Balancing complexity and clarity—towards clinician-ready antibiotic resistance prediction models

**DOI:** 10.1093/bioinformatics/btaf556

**Published:** 2025-10-01

**Authors:** Dickson Aruhomukama

**Affiliations:** Department of Medical Microbiology, College of Health Sciences, Makerere University (MakCHS), Kampala, 7072, Uganda; Department of Global Health Security, The Infectious Diseases Institute (IDI), MakCHS, Kampala, 22418, Uganda

## Abstract

**Motivation:**

The escalating challenge of antibiotic resistance (ABR) demands clinician-ready machine learning models that are not only accurate but interpretable.

**Results:**

By treating resistance genes as independent features and augmenting them with curated single-nucleotide polymorphisms and contextual markers, this approach delivers scalable, transparent predictions aligned with clinical decision-making needs.

**Availability and implementation:**

Not applicable.

The escalating crisis of antibiotic resistance (ABR) necessitates innovative, scalable solutions to improve detection and prediction using genomic data. Machine learning (ML) is increasingly being used to predict ABR from pathogen genomes (Kim et al. 2022, Ren et al. 2022). Among modeling approaches, treating ABR genes as independent predictors strikes a balance between biological complexity and clinical utility. While critics note that this may oversimplify gene interactions (Kim et al. 2022), the benefits in interpretability, scalability, and computational efficiency (Ren et al. 2022) are compelling for real-world healthcare. Clinicians require ABR prediction tools that are both accurate and transparent, aligning with decision-making frameworks under time constraints.

The strength of independent-gene models lies in interpretability, scalability, and computational efficiency (Ren et al. 2022). By treating resistance genes as discrete features, these models enable clinicians to directly link genetic markers to predictions. For example, the presence of blaTEM genes correlates strongly with β-lactam resistance in Enterobacteriales (Schechter et al. 2018), providing actionable insights without decoding complex epistatic effects. Similarly, mecA indicates methicillin resistance in *Staphylococcus aureus* and guides therapy. These presence/absence markers align with clinical reasoning and model logic can be visualized through decision trees or feature importance rankings, promoting transparency. In emergencies, such interpretability is critical for rapid, evidence-based decisions.

Technical simplicity affords major practical benefits. Modeling gene-gene interactions or complex nonlinear effects requires exponentially more data, computing power, and often risks overfitting (Bai et al. 2024). In contrast, treating genes independently drastically reduces dimensionality, yielding models that train faster, use fewer resources, and can run on standard hardware (Bai et al. 2024). For example, traditional ML algorithms like logistic regression or decision trees typically train in minutes on CPUs (Central Processing Units), whereas deep neural networks may require powerful GPUs (Graphics Processing Units) and much longer training times. Independent-gene models thus adapt easily to new features: adding a novel resistance gene simply adds one binary input, avoiding costly retraining (Okser et al. 2014). This computational efficiency supports deployment even in LICs (low-income countries) with limited computational infrastructure.

Critics rightly observe that ABR is influenced by biological context—MGEs (mobile genetic elements), HGT (horizontal gene transfer), and regulatory variants can affect resistance. We can address this by encoding contextual markers alongside genes. For example, features can include indicators of integrase genes, specific plasmid replicons, or co-occurrence scores for groups of genes on the same element. This hybrid approach preserves interpretability: a clinician can understand that “Gene X plus integrase present” indicates elevated risk. For instance, a model might flag the combination of blaCTX-M with and integron integrase (Intl1) as higher risk for multidrug resistance than blaCTX-M alone, capturing key interactions without using a fully opaque model (Nguyen et al. 2021).

Beyond gene presence, we incorporate expert-curated SNPs (single-nucleotide polymorphisms) known to impact resistance. Examples include mutations in drug targets (like specific gyrA codon changes for fluoroquinolone resistance), regulatory SNPs (such as promoter mutations increasing AmpC expression), and variants of resistance genes that alter function. Each curated SNP is encoded as a binary (present/absent) or simple categorical feature. For example, we encode the Proline-to-Serine substitution at position 167 in blaCTX-M (known to enhance ceftazidime hydrolysis) as a distinct feature. To avoid feature explosion, we include only SNPs that are tightly linked to resistance loci and have experimental validation. This targeted inclusion of SNP features refines predictions without obscuring them: clinicians can interpret these markers as “resistance amplifiers” or modifiers in a way that matches mechanistic knowledge (Yang and Wu 2022).

Transparency is essential for clinical trust. Machine learning models should produce outputs aligned with biological reasoning. We can leverage tools like SHAP (SHapley Additive exPlanations, a game theoretic approach to explain the output of any ML model) to quantify each feature’s influence on a prediction (e.g. comparing the contribution of blaCTX-M-15 versus a gyrA mutation). Similarly, the model’s logic can be presented as simple decision rules: for example, if mecA is present, predict methicillin resistance; otherwise, check for blaZ. Such clear rules let clinicians follow the reasoning. When a model makes a surprising prediction, laboratory staff can audit the key features (for instance, a high SHAP weight on an otherwise obscure AmpC promoter SNP) and validate with phenotypic tests. By keeping models interpretable, we align AI (artificial intelligence) predictions with the clinician’s mental model of resistance mechanisms.

Different modeling strategies trade off accuracy, complexity, and interpretability. Interaction-aware ML methods or deep learning can capture subtle patterns across many genes. However, they typically demand much more data and computational resources, and their decision processes are largely opaque to users. Independent-gene models, in contrast, run efficiently on modest hardware and yield immediately understandable decisions. In practical terms, a deep learning model might achieve only marginal accuracy gains over a simpler model while requiring expensive GPUs and specialized expertise. In lieu of numeric benchmarking, we qualitatively note that advanced hybrid approaches (e.g. combining an interpretable model with a small black-box component) can sometimes slightly boost performance but at the cost of complexity and reduced transparency. Especially in clinical and public-health settings, the slight performance gains of “black-box” methods often do not outweigh their drawbacks on interpretability, updating, and deployment (Weiss and Indurkhya 1995, Wang and Lin 2021, Malla and Banka 2023).

All features (genes, SNPs, MGEs) are encoded in user-friendly ways: mostly binary indicators or simple categories. In practice, we construct a feature matrix where each column represents a curated gene, SNP or contextual marker, and rows are isolates. To keep models sparse and interpretable, we regularize heavily. For example, in a penalized logistic regression, we use L1 or L2 regularization to shrink irrelevant feature weights to zero, effectively selecting only the most predictive markers. In tree-based models, we limit depth and require minimum evidence for splits. When multiple SNPs affect the same gene, we may group them or include only the single most impactful mutation per gene, avoiding redundant features. Interaction terms (e.g. gene × integrase) are added only if there is strong biological justification. This disciplined feature integration ensures the model remains modular: each part of the model can be individually examined, helping clinicians to validate and trust the tool.

Several real-world challenges complicate implementation. First, genotype-phenotype gaps persist: not every gene presence means expressed resistance, and phenotypic outcomes can be multifactorial. For example, certain resistance determinants (cryptic blaAmpC variants or some qnr genes) may be present without causing strong resistance, adding noise to predictions. Factors like variable gene expression, compensatory mutations, and epistatic interactions can also weaken correlations. Second, data quality is variable. Effective modeling requires high-quality, diverse genomic and phenotypic data. In practice, sequencing coverage can be uneven, assemblies incomplete, and laboratory phenotypic protocols differ. Changing clinical breakpoints between standards (e.g. EUCAST (European Committee on Antimicrobial Susceptibility Testing) versus CLSI (Clinical and Laboratory Standards Institute)) further complicates labels. Third, models require continual updating. New resistance genes and variants emerge frequently: without regular retraining on fresh data, models can quickly become outdated. Establishing pipelines for continual data integration and model validation is necessary but demanding. Finally, resource constraints in some settings pose barriers: limited access to sequencing, computational hardware, and trained personnel can hamper adoption. Designing lightweight, automated tools with clear interfaces is critical to support practical deployment in LICs.

Treating ABR genes as independent predictors is not a compromise but a strategic foundation for scalable, clinician-ready tools. When augmented with curated SNPs and contextual markers, this approach yields modular models that balance biological insight with practical utility. While no model captures every nuance, our framework prioritizes actionable insights that clinicians can understand and trust. As ABR outpaces drug development, such pragmatic modeling innovations are not just relevant—they are essential.

## Data Availability

Not applicable.
